# Remnant cholesterol, iron status and diabetes mellitus: a dose–response relationship and mediation analysis

**DOI:** 10.1186/s13098-024-01304-0

**Published:** 2024-03-12

**Authors:** Xiangming Hu, Yan Lin, Allison A. Appleton, Weimian Wang, Bingyan Yu, Langping Zhou, Guang Li, Yingling Zhou, Yanqiu Ou, Haojian Dong

**Affiliations:** 1 Department of Cardiology, Guangdong Provincial People’s Hospital (Guangdong Academy of Medical Sciences), Southern Medical University, Guangzhou, Guangdong China; 2 Department of Cardiology, National Center for Cardiovascular Diseases, Fuwai Hospital, Chinese Academy of Medical Sciences and Peking Union Medical College, Beijing, China; 3 Shantou University Medical College, Shantou, Guangdong China; 4 Department of Epidemiology and Biostatistics, School of Public Health, University at Albany, State University of New York, 1 University Place, Rensselaer, NY USA; 5 The Second School of Clinical Medicine, Southern Medical University, Guangzhou, Guangdong China; 6 Department of Cardiology, Baoan District Central Hospital, Shenzhen, Guangdong China

**Keywords:** Remnant cholesterol, Iron, Diabetes mellitus, Mediation

## Abstract

**Background:**

Remnant cholesterol (RC) is recognized as a risk factor for diabetes mellitus (DM). Although iron status has been shown to be associated with cholesterol metabolism and DM, the association between RC, iron status, and DM remains unclear. We examined the relationship between RC and iron status and investigated the role of iron status in the association between RC and DM.

**Methods:**

A total of 7308 patients were enrolled from the China Health and Nutrition Survey. RC was calculated as total cholesterol minus low-density lipoprotein cholesterol and high-density lipoprotein cholesterol. Iron status was assessed as serum ferritin (SF) and total body iron (TBI). DM was ascertained by self-reported physician diagnosis and/or antidiabetic drug use and/or fasting plasma glucose ≥ 126 mg/dL and/or glycated haemoglobin ≥ 6.5%. General linear models were used to evaluate the relationships between RC and iron status. Restricted cubic splines were used to assess the association between RC and DM. Mediation analysis was used to clarified the mediating role of iron status in the association between the RC and DM.

**Results:**

The average age of the participants was 50.6 (standard deviation = 15.1) years. Higher RC was significantly associated with increased SF (β = 73.14, SE = 3.75, 95% confidence interval [CI] 65.79–80.49) and TBI (β = 1.61, SE = 0.08, 95% CI 1.44–1.78). J-shape relationships were found in the association between RC levels with DM, as well as iron status with DM. Significant indirect effects of SF and TBI in the association between RC and DM were found, with the index mediated at 9.58% and 6.37%, respectively.

**Conclusions:**

RC has a dose–response relationship with iron status. The association between RC and DM was mediated in part by iron status. Future studies are needed to confirm these findings and further clarify the underlying mechanism.

**Supplementary Information:**

The online version contains supplementary material available at 10.1186/s13098-024-01304-0.

## Introduction

Diabetes mellitus (DM) has become one of the most significant public health problems with high morbidity and mortality rate [1]. According to the 2021 International Diabetes Federation, there are 537 million adults living with DM worldwide, and the number is expected to reach 643 million by 2030 [2].

Traditional risk factors for DM, including aging, family history, obesity, dyslipidemia, physical inactivity, lifestyle, and heavy metals exposure have been extensively studied [3, 4]. Among them, dyslipidemia-related hyperglycemia is highly focused in recent years [5]. There is growing evidence that dyslipidemia may not only contribute to glucose metabolism disorders, but may also be associated with the development of DM [6, 7]. Remnant cholesterol (RC) is recognized as the cholesterol component in triglyceride-rich lipoproteins (TRLs) that consists of very low-density lipoproteins, intermediate-density lipoproteins, and chylomicron remnants [8]. Prospective studies indicated that increased RC levels serves as an independent risk factor for DM [9–11]. A study involving 15,464 individuals who underwent health examinations and were followed up for a median of 6.1 years found that elevated RC was associated with an increased risk of DM after adjusting for demographic information, lifestyle, and liver function [9]. In addition, RC was found to have the greatest predictive value of DM compared to other lipid indices [9]. Huh et al. conducted a cohort study including over 8.48 million Korean adults found a significant association between RC and incident DM over a follow-up period of 9.3 years, further taking chronic kidney disease and lipid-lowering drugs use into account [10]. Moreover, another multicenter study involving 36684 individuals indicated that RC was significantly associated with DM, even when levels of other lipids were controlled within the ranges recommended by guidelines [11].

Iron status and iron-regulated pathways affect lipid and glucose metabolism [12]. While iron is an essential mineral that implicated in many important physiological processes, high iron levels are highly associated with increased metabolic disease risk [13–15]. In addition to the observed abnormal metabolism of lipids related to iron load, there is also an association between iron storage within the physiological range and the risk of DM [16]. Evidence from recent epidemiological studies have shown that rising serum ferritin (SF) levels were significantly associated with higher cholesterol levels [17–19]. Furthermore, iron loading could induce the oxidation of cholesterol and elicit oxidative stress–mediated damage [20, 21]. The cholesterol-related oxidative stress has been shown to be related islet β cell dysfunction, leading to the progression of DM [5]. RC is an important conponent of the cholesterol profile, but there are still no studies to varify its relationship with iron status. Given the regulatory role of iron status on cholesterol homeostasis [21, 22], and the established correlation between RC and DM [9–11], it is imperative to further clarify the relationship between RC, iron status and DM.

We hypothesize that the levels of RC are positively correlated with iron status and the prevalence of DM, and iron status partially mediates in the association between RC and DM. To consider these associations, we leverage data from the China Health and Nutrition Survey (CHNS), a large population-based study of nutritional factors and disease.

## Methods

### Study design and population

The CHNS is an ongoing community-based longitudinal cohort study in China that enrolled more than 12,000 individuals in approximately 9 provinces. Health information was obtained via questionnaires, household surveys, and biospecimen collection. Study details and protocols have been previously published elsewhere [23]. The CHNS was approved by the Institutional Review Board of the University of North Carolina at Chapel Hill, the National Institute for Nutrition and Food safety at China Center for Disease Control and Prevention, and the Human and Clinical Research Ethics Committee of the China-Japan Friendship Hospital.

**Fig. 1:**
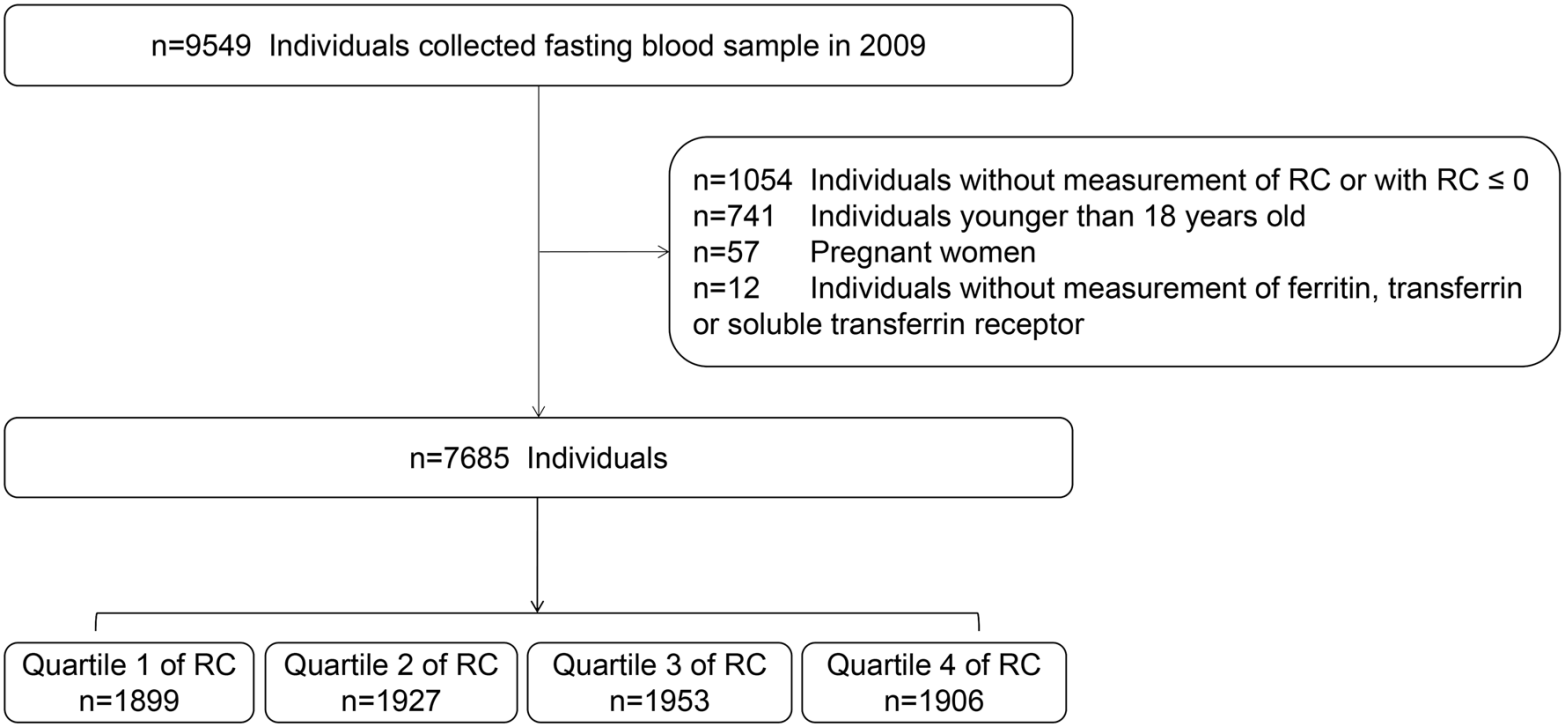
Study flowchart. *RC* Remnant cholesterol

Within the research Waves of the CHNS, blood test was only available in Wave 2009 among 9549 participants. Of these, 1054 participants without RC measurements, 57 pregnant women, 741 participants who were under 18 years old, and 12 participants missing information about iron status markers were excluded in this study. Thus, the present analysis included 7685 participants (Fig. 1). Baseline characteristics of included versus excluded participants are also listed (Additional file 1: Table S1).

### Measures and definitions

RC (mmol/L) was calculated as total cholesterol (TC) (mmol/L) minus low-density lipoprotein cholesterol (LDL-C, mmol/L), minus high-density lipoprotein cholesterol (HDL-C, mmol/L) [24]. Iron status was assessed as serum ferritin (SF) and total body iron (TBI) [25]. The concentration of TBI was measured using ferritin and soluble transferrin receptor (sTfR), and was calculated as the following formula: (TBI =  −  [log(sTfR/SF) − 2.28229]/0.1207) [26].

Prior to blood sample collection, individuals were asked to maintain a normal lifestyle for at least 3 days and then fasted for 8–12 h. Transferrin and sTfR were detected by Siemens BNP (Siemens, Germany) via nephelometry. Ferritin was measured by Gamma counter XH-6020 (North Institute of Bio-Tech, China) via radioimmunology. TC, HDL-C, and LDL-C were measured using the CHOD-PAP method, and the polyethylene glycol-modified enzyme method, respectively, by determiner regents [Kyowa Medex Co., Ltd, Tokyo, Japan]. Fasting blood glucose (FBG) and triglyceride (TG) were measured with the GOD-PAP method by determiner regents (Randox Laboratories Ltd, UK for FBG, and Kyowa Medex Co., Ltd, Tokyo, Japan for TG). Creatinine was measured with the picric acid method by determiner regents (Randox Laboratories Ltd, UK). All lipid and creatinine measures were on the Hitachi 7600 automated analyzer (Hitachi Inc., Tokyo, Japan). White blood cells (WBCs) and hemoglobin was measured with a Beckman Coulter LH751 (Beckman Coulter, USA). High-sensitivity C-reactive protein (Hs-CRP) was measured using the immunoturbidimetric method by a Hitachi 7600 (Denka Seiken, Japan). Routine blood test was on-site test and all local laboratories were asked to provide their Levey-Jennings chart for one month. Other samples were analyzed in a national central lab in Beijing (medical laboratory accreditation certificate ISO 15189: 2007) with strict quality control. Homeostatic model assessment of insulin resistance (HOMA-IR) was calculated by: FBG (mmol/L) × fasting insulin level (mIU/L) /22.5.

DM was defined according to medical diagnosis and/or receiving treatment for DM according to questionnaire responses to the following items: “Has a doctor ever told you that you have DM?” and “Have you used any of the following treatments such as special diets, weight control, oral medications, insulin injections, traditional Chinese medicine, home remedies, or qigong/spiritual therapy)?” In addition, an additional criterion (fasting blood glucose ≥ 126 mg/dL, and/or HbA1c ≥ 6.5% according to the American Diabetes Association criteria was also applied for DM screening [27]. Diagnosis of anemia based on hemoglobin concentration (< 120 g/L in men, < 110 g/L in women).

### Assessment of covariates

Covariates that could possibly confound the associations between RC, iron, and DM were included in analysis and are described in turn below. Height and weight were measured while the participants were wearing light clothing without shoes by the study staff. Body mass index (BMI) was calculated by weight (kg)/height (m)^2^. Renal function assessed via the estimated glomerular filtration rate (eGFR) calculated using the Chronic Kidney Disease (CKD) Epidemiology Collaboration (CKD-EPI) equation [28]. CKD was diagnosed based on an eGFR of < 60 mL/min^1^/1.73 m^2^. Urban or rural residence, occupation (farmer [including fishermen and hunters]/non-farmer), health behaviors (smoking and alcohol consumption) and education levels (upper middle school and above/ Junior high school or below) were self-reported. Smoking was defined as any previous smoking (yes/no), and alcohol consumption was defined as greater than three times per week (yes/no). History of hypertension was defined by medical diagnosis as reported to the following questionnaire item: “Has a doctor ever told you that you have hypertension?” Cardiovascular disease was defined as having one of the following conditions: coronary artery disease, stroke, or transient ischemic attack. Antidiabetic drug use included oral medications and insulin injections. Energy intake, carbohydrate intake, fat intake and protein intake per day were all calculated by multiplying the intake of each food by the standard serving size (100 g) from the average self-reported dietary intake for 3 days. Participant age and sex were also examined.

### Statistical analysis

All participants were divided into four groups according to quartiles of RC levels. Continuous variables were presented as mean ± standard deviation for normal distributions or medians and interquartile range (IQR, 25–75%) for skewed distributions, and categorical variables were expressed as frequency (percentages). The generalized linear regression analysis and Cochran-Armitage trend χ^2^ test were employed to test for trend across RC quartiles for continuous and categorical variables, respectively. Then, we used a general linear model to evaluate the associations between RC and iron status markers by β-coefficient and 95% confidence intervals (95% CIs). According to the STROBE recommendation, we simultaneously showed the results from unadjusted, minimally adjusted and fully adjusted analyses [29]. Potential confounders that were significant in the univariate analysis or clinically important were included for model adjustment. We also developed directed acyclic graphs (DAGs) for the selection of covariates (Additional file 1: Figs. S1, S2). For the association between RC and iron status, the confounders included age, sex, BMI, residence, occupation, education, smoking, alcohol consumption, eGFR, LDL-C, HDL-C, average energy intake, average carbohydrate intake, average fat intake, and average protein intake. Subgroup analyses (including age, sex, BMI, residence, occupation, education, smoking, CKD, anemia and alcohol consumption) were performed using stratified linear regression models. Moreover, to explore the possible dose–response relationships between RC, iron status markers and DM prevalence, logistic regression assigned to all subjects was performed by incorporating a restricted cubic spline (RCS) function with odds ratios (ORs) and 95% CIs, adjusting for age, sex, BMI, residence, occupation, education, smoking, alcohol consumption, eGFR, LDL-C, HDL-C, antidiabetic drug, average energy intake, average carbohydrate intake, average fat intake, and average protein intake. Four knots at the 5th, 35th, 65th and 95th percentiles were set for the RCS. Finally, the causal steps approach based on R package “mediation” was conducted to investigate the effect of RC on DM partially mediated through SF and TBI, and Sobel test was performed to avoid missing any significant results [30]. Adjusting for all covariates in Model 3, bootstrapping method with 10,000 repeats was used to estimate the 95% CI of indirect (mediated) effects. Mediation was confirmed if the bias-corrected 95% CI for the indirect effect did not include zero. Additionally, sensitivity analyses were performed to specifically assess the association of RC and iron status: (1) further considering TG and hs-CRP as covariate, and (2) excluding participants without covariates.

The proportion of missing data in the analysis sample was not more than 2%. Missing data was interpolated by the method of the last observation carried forward, or the mean value of continuous variables and the median value of skewed variables. Associations where p < 0.05 (two-sided) were considered to be statistically significant. We performed all analyses with Stata 15.0 and R (version 4.0.2).

## Results

### Baseline characteristics

The average age of the participants was 50.6 ± 15.1 years old, and the median RC levels were 0.37 mmol/L (IQR, 0.20–0.65). Table 1 summarized the demographic and clinical characteristics of the study population according to quartiles of RC. There were no significant differences in alcohol consumption and average energy intake according to RC quartile. Compared with the lowest RC level group, people in the other three groups (quantile, Q2-Q4) were more likely to be older, male and smoker, more from urban areas, had a higher BMI and level of education. In terms of bio-assay analysis, high RC level accompanied by higher levels of TC, TG, apolipoprotein-B, hemoglobin, WBC, hemoglobin, Hs-CRP and lower levels of HDL-C and eGFR. As for iron status markers, higher levels of SF, transferrin, TBI and lower levels of sTfR were shown in the highest quartile of RC compared with the lowest quartile.

**Table 1:** Baseline characteristics according to quartiles of RC

Remnant cholesterol	Q1 (0.01–0.19) n = 1899	Q2 (0.20–0.36) n = 1927	Q3 (0.37–0.65) n = 1953	Q4 (0.66–8.68) n = 1906	P-value
Age, year	49.68 ± 15.39	50.21 ± 15.96	51.06 ± 15.34	51.53 ± 13.79	< 0.001
Male sex	836 (44.02%)	866 (44.94%)	935 (47.88%)	1038 (54.46%)	< 0.001
BMI, kg/m^2^	22.45 ± 3.14	22.85 ± 3.32	23.71 ± 3.51	24.95 ± 3.49	< 0.001
Residence					0.002
Urban	604 (31.81%)	593 (30.77%)	671 (34.36%)	676 (35.47%)	
Rural	1295 (68.19%)	1334 (69.23%)	1282 (65.64%)	1230 (64.53%)	
Upper middle school and above	400 (21.06%)	452 (23.46%)	482 (24.68%)	496 (26.02%)	< 0.001
Farmer	1038 (54.66%)	973 (50.49%)	928 (47.52%)	866 (45.44%)	< 0.001
Smoking	538 (28.33%)	566 (29.37%)	619 (31.69%)	690 (36.20%)	< 0.001
Alcohol consumption	263 (13.85%)	208 (10.79%)	235 (12.03%)	300 (15.74%)	< 0.001
TC, mmol/L	4.67 ± 0.93	4.72 ± 0.96	4.85 ± 0.99	5.19 ± 1.05	< 0.001
TG, mmol/L	0.88 ± 0.39	1.15 ± 0.56	1.61 ± 0.73	3.42 ± 2.09	< 0.001
LDL-C, mmol/L	2.97 ± 0.87	2.98 ± 0.91	3.01 ± 0.93	2.76 ± 1.00	< 0.001
HDL-C, mmol/L	1.59 ± 0.35	1.47 ± 0.34	1.35 ± 0.31	1.15 ± 0.29	< 0.001
RC, mmol/L	0.11 (0.06–0.16)	0.27 (0.23–0.32)	0.48 (0.42–0.56)	1.01 (0.80–1.43)	< 0.001
WBCs, 10^9^/L	5.98 (1.78)	6.22 (1.94)	6.46 (1.96)	6.74 (2.20)	< 0.001
Hemoglobin, g/L	138.77 ± 20.36	139.56 ± 19.73	141.81 ± 20.60	145.90 ± 20.00	< 0.001
Hs-CRP, mg/L	1.00 (0.00–2.00)	1.00 (0.00–2.00)	1.00 (1.00–3.00)	2.00 (1.00–3.00)	< 0.001
eGFR, ml/min/1.73m^2^	80.80 ± 16.51	79.44 ± 17.34	78.09 ± 17.24	78.24 ± 16.63	< 0.001
HOMA-IR	2.76 ± 5.34	3.19 ± 6.63	3.90 ± 6.51	5.69 ± 10.15	< 0.001
SF, ng/mL	62.42 (29.48–109.88)	72.26 (37.89–130.10)	89.58 (46.54–162.41)	119.61 (64.79–225.20)	< 0.001
TRF, mg/dL	277.00 (249.00–313.00)	277.00 (246.00–313.00)	280.00 (248.00–315.00)	293.00 (261.00–328.00)	< 0.001
sTfR, mg/L	1.34 (1.12–1.66)	1.33 (1.08–1.65)	1.34 (1.07–1.69)	1.32 (1.05–1.63)	0.003
TBI, mg/kg	32.77 (29.88–35.02)	33.37 (30.75–35.66)	34.04 (31.57–36.45)	35.18 (32.69–37.77)	< 0.001
Average energy intake, kcal/day	2069.99 (1656.31–2519.77)	2051.42 (1659.43–2499.52)	2091.31 (1688.34–2502.86)	2090.93 (1700.67–2485.68)	0.353
Average carbohydrate intake, g/day	284.80 (223.85–360.26)	281.48 (222.52–353.66)	282.23 (224.64–348.81)	279.61 (222.01–343.51)	0.036
Average fat intake, g/day	67.47 (48.24–92.00)	67.93 (48.04–92.98)	71.56 (50.83–95.10)	70.76 (50.31–95.10)	0.004
Average protein intake, g/day	61.94 (49.55–76.98)	61.27 (49.01–77.44)	63.47 (50.47–78.39)	63.87 (51.01–79.77)	< 0.001
Anemia	116 (6.11%)	112 (5.81%)	108 (5.53%)	69 (3.62%)	< 0.001
DM	101 (5.32%)	150 (7.78%)	220 (11.26%)	402 (21.09%)	< 0.001
Hypertension	165 (8.69%)	206 (10.69%)	294 (15.05%)	362 (18.99%)	< 0.001
Cardiovascular disease	41 (2.16%)	31 (1.61%)	57 (2.92%)	57 (2.99%)	0.015
Antidiabetic drug use	13 (0.68%)	8 (0.42%)	18 (0.92%)	20 (1.05%)	0.064

*BMI* Body mass index, *DM* Diabetes mellitus, *eGFR* Estimated glomerular filtration rate, *HDL-C* High-density lipoprotein cholesterol, *HOMA-IR* Homeostatic model assessment of insulin resistance, *Hs-CRP* High-sensitivity C-reactive protein, *LDL-C* Low-density lipoprotein cholesterol, *RC* Remnant cholesterol, *SF* Serum ferritin, *sTfR* Soluble transferrin receptor, *TC* Total cholesterol, *TBI* Total body iron, *TG* Triglyceride, *TRF* Transferrin, *WBCs* White blood cells

### The relationship between remnant cholesterol and iron status markers

**Fig. 2:**
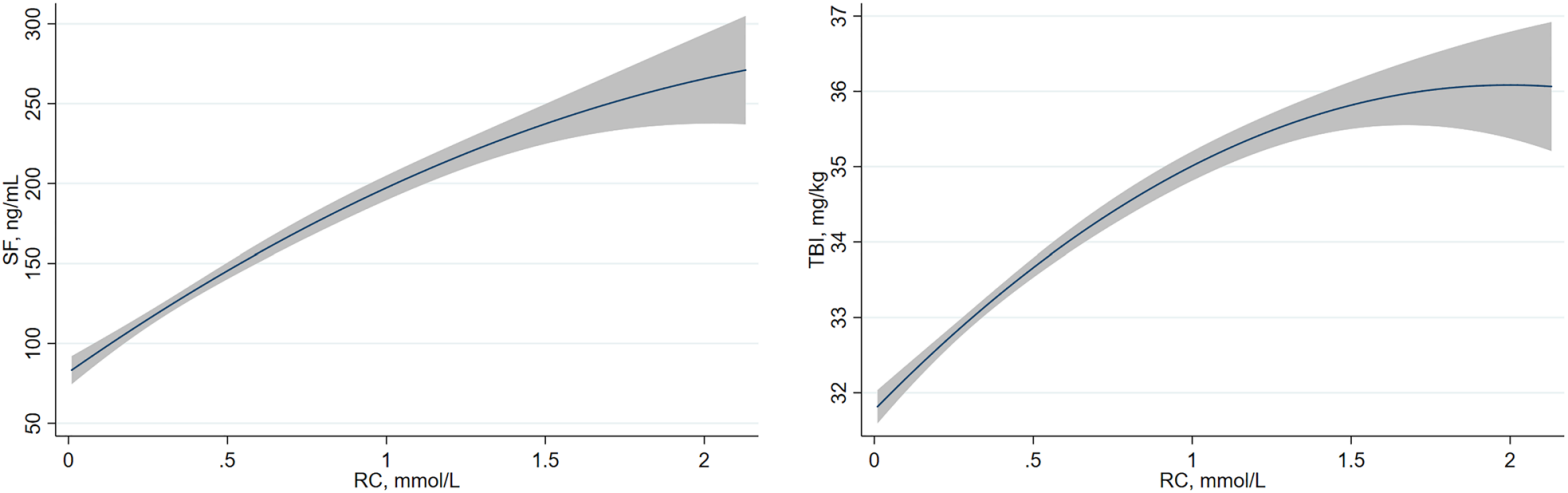
Association of RC with iron state. Values outside the 97.5th percentile of RC are not included. *RC* Remnant cholesterol, *SF* Serum ferritin, *TBI* Total body iron

Generalized linear regression models testing the association between SF and TBI with RC are shown in Table 2. RC was significantly positively associated with iron status markers in fully adjusted models (β = 73.14, SE = 3.75, 95% CI 65.79–80.49, p < 0.001 for SF; β = 1.61, SE = 0.08, 95% CI 1.44–1.78, p < 0.001 for TBI). Also, compared to the lowest quartile, participants in the highest quartile of RC levels had increased levels of SF (β = 86.74, SE = 6.47, 95% CI 74.05–99.43, p for trend < 0.001) and TBI (β = 2.21, SE = 0.15, 95% CI 1.92–2.50, p for trend < 0.001) in the fully adjusted models. In the different subgroups (age, sex, BMI, residence, occupation, education, smoking, alcohol consumption, CKD and anemia), RC was still significantly associated with SF and TBI in most stratum (Additional file 1: Tables S2, S3). In order to further characterize the relationship between RC and iron status markers, the dose–response relationships are presented in Fig. 2. Overall, the curves show that as RC increased, SF and TBI increased rapidly, and then began to flatten at the highest levels of RC.

**Table 2:** General linear regression models for the associations between remnant cholesterol and iron status

	Model 1	Model 2	Model 3
SF, ng/ml			
Each 1 mmol/L increase in RC	82.63 (75.85–89.41)	65.97 (59.35–72.58)	73.14 (65.79–80.49)
Q1 (0.01–0.19)	Reference	Reference	Reference
Q2 (0.20–0.36)	18.87 (7.02–30.72)	15.27 (4.08–26.45)	15.03 (3.76–26.29)
Q3 (0.37–0.65)	51.73 (39.92–63.54)	39.38 (28.13–50.62)	38.48 (26.90–50.05)
Q4 (0.66–8.68)	113.53 (101.65–125.42)	86.15 (74.53–97.78)	86.74 (74.05–99.43)
P for trend	< 0.001	< 0.001	< 0.001
TBI, mg/kg			
Each 1 mmol/L increase in RC	1.91 (1.74–2.08)	1.42 (1.26–1.57)	1.61 (1.44–1.78)
Q1 (0.01–0.19)	Reference	Reference	Reference
Q2 (0.20–0.36)	0.68 (0.39–0.97)	0.57 (0.32–0.83)	0.57 (0.31–0.83)
Q3 (0.37–0.65)	1.56 (1.27–1.85)	1.19 (0.93–1.44)	1.16 (0.89–1.42)
Q4 (0.66–8.68)	2.96 (2.67–3.25)	2.15 (1.89–2.42)	2.21 (1.92–2.50)
P for trend	< 0.001	< 0.001	< 0.001

Model 1: unadjusted;

Model 2: adjusted for age, sex and BMI;

Model 3: adjusted for age, sex, BMI, residence, occupation, education, smoking, alcohol consumption, eGFR, LDL-C, HDL-C, average energy intake, average carbohydrate intake, average fat intake, and average protein intake

*RC* Remnant cholesterol, *SF* Serum ferritin, *TBI* Total body iron

### Remnant cholesterol, iron status biomarkers and diabetes mellitus

**Fig. 3:**
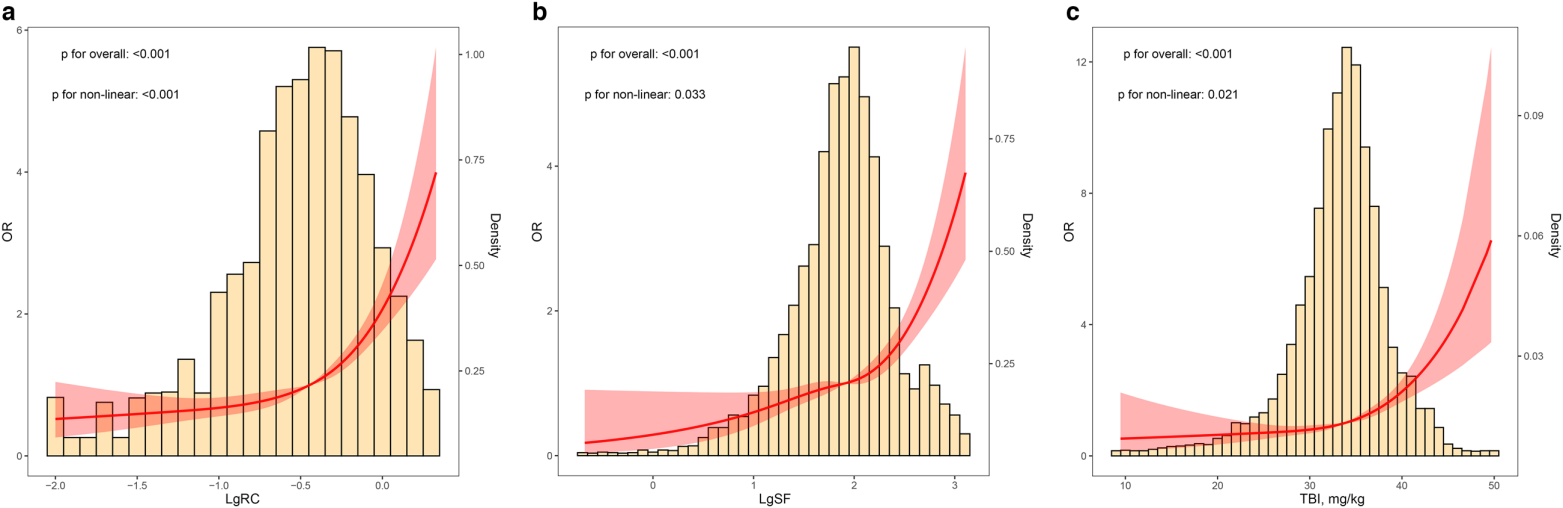
The restricted spline curve for the association between RC, iron status markers and DM. The histogram of the distribution of lgRC, lgSF, and TBI, and the RCS for the associations between lgRC, lgSF, TBI, and DM with four knots. The red line and shadow area represent odds ratios (ORs, solid lines) and 95% confidence intervals (CIs) after multivariable adjustment for age, sex, BMI, residence, occupation, education, smoking, alcohol consumption, eGFR, LDL-C, HDL-C, antidiabetic drug, average energy intake, average carbohydrate intake, average fat intake, and average protein intake based on the RCS models. RC and SF were lg-transformed and the concentration of lgRC and lgSF were -0.45 and 1.9 [OR = 1] as the reference concentration, respectively. While the concentration of TBI was 34 mg/kg [OR = 1] as the reference concentration. Values outside the 97.5th percentile of RC are not included. *RC* Remnant cholesterol, *OR* Odds ratio, *CI* Confidence interval, *SF* Serum ferritin, *TBI* Total body iron

In wave 2009, a total of 873 (11.4%) participants had DM. As shown in Fig. 3, multivariable-adjusted spline regression models indicated that lgRC, lgSF, and TBI levels were non-linearly related to DM (p for non-linearity: < 0.05), with the shape of the relationship demonstrating a “J” shape. And the cut-off values of RC, SF, and TBI associated DM were about 0.35 mmol/L, 79.5 ng/mL, and 34 mg/kg, respectively.

### Mediation analysis

**Fig. 4:**
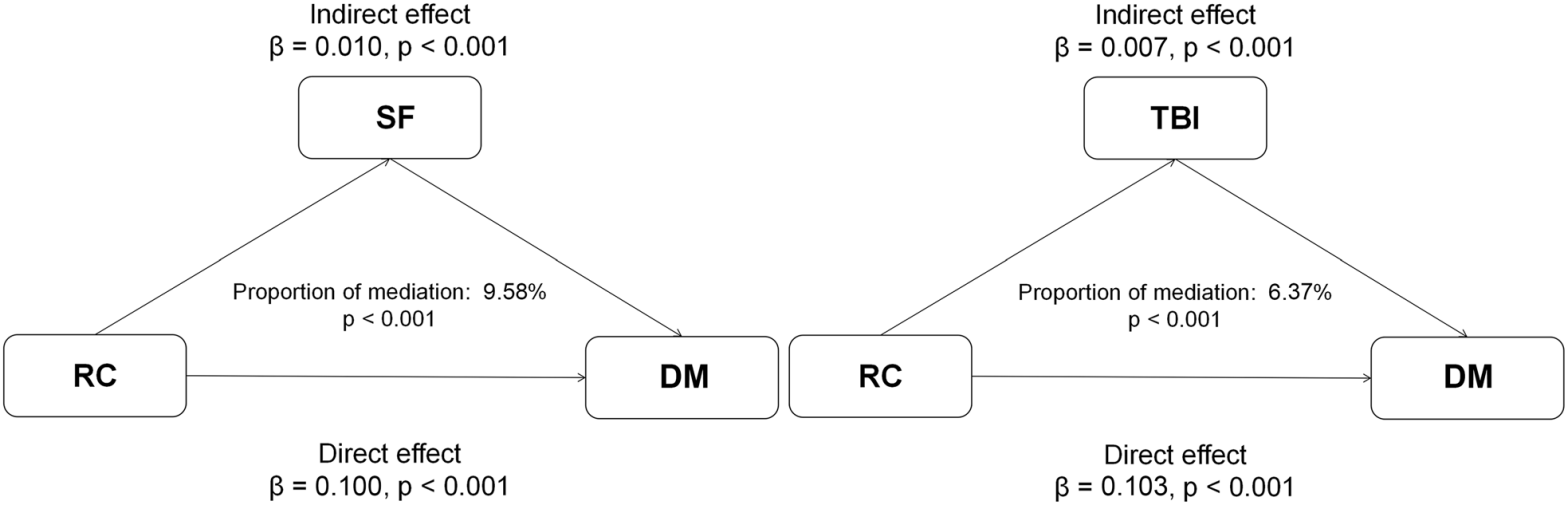
Mediation analysis of the association between RC and DM. *DM* Diabetes mellitus, *RC* Remnant cholesterol, *SF* Serum ferritin, *TBI* Total body iron

Potential mediation effects of iron status in the association between RC and DM is presented in Fig. 4. A significant positive indirect effect of RC associated with DM through iron status was observed, and the proportion mediated effect was 9.58% for SF (p < 0.001) and 6.37% for TBI (p < 0.001).

### Sensitivity analysis

To further test the robustness of the relationship of RC and iron status markers, we carried out sensitivity analysis, where the TG and hsCRP were adjusted. Patients in the highest quartile of RC were still associated with an increased levels of SF and TBI when considering TG (Additional file 1: Table S4) and hs-CRP (Additional file 1: Table S5). After excluding patients with missing baseline covariates, the results were consistent with the primary findings (Additional file 1: Table S6 and Additional file 1: Fig. S3).

## Discussion

To our knowledge, this is the first study to investigate the relationship between RC and iron status, as well as to explore the role of iron status in the relationship between RC and DM. The present study indicated positive associations between RC, SF and TBI in a large Chinese population study, independent of potential confounders. Higher levels of RC, SF, and TBI were associated with increased prevalence of DM. Iron status partially mediated the relationship between RC and DM.

Previous studies indicated a correlation between cholesterol levels and iron status [17, 18, 31]. Sun et al. included 3289 adults aged 50 to 70 and analyzed the relationship between iron status and metabolic risk factors [17]. They found a significant positive correlation between SF and LDL-C, and a significant negative correlation with HDL-C. Kim et al. studied the relationship between SF levels and dyslipidemia among 1879 adolescents from Korean National Health and Nutrition Examination Survey IV, revealing that SF levels were negatively correlated with HDL-C [18]. However, these studies didn’t specifically focused on RC. Evidence suggested that iron status had a significant interaction with cholesterol metabolism in the liver, which may affect the levels of circulating cholesterol [31]. Although the relationship between traditional cholesterol and iron status markers has been disclosed, there is a paucity of research work in the association between RC and iron status. Our study addressed this gap, providing evidence of the relationship between RC and iron status. In our study, we observed that RC levels were positively correlated with SF and TBI, the two indicators of iron status, after further consideration of nutrient intake. And this relationship was also nearly identical across all subgroups. As systemic inflammation has been related to iron overload, we also adjusted for hs-CRP in the sensitivity analysis and the results remained consistent. The association of RC and iron status persisted when TG were taken into account, which suggested that this association was independent of the TG component.

RC and iron status as risk factors for DM have been widely reported [7–9, 32–35]. The correlation between RC and DM remained consistent even after adjusting for demographic characteristic, lifestyle, comorbidities, and lipid-lowering medications [7–9]. In our study, we identified that the threshold of RC for its correlation with DM was 0.35 mmol/L. The thresholds for RC related to DM reported across different studies showed considerable variation [9, 32, 33], possibly due to differences in study population, RC measurement, and definition of DM. As for the association between iron status and DM, Sun et al. found a significant correlation between SF and DM after adjusting for traditional risk factors, inflammatory and adipokines [17]. Feng et al. revealed a nonlinear association between SF and DM in a study of 1145 women of childbearing age, even after adjusting for blood lipids, hemoglobin, and α-acid glycoprotein [34]. Notably, they identified a threshold level of 101.4 ng/mL for SF, which was similar to our study. Even an increase in SF within the physiological range was associated with insulin resistance [35], which supported the findings in the present study.

The role of iron in regulating cellular lipid metabolism has been established, indicating that iron status was associated with the biological function of cholesterol [36]. A Mendelian randomization study revealed a causal effect of iron status on cholesterol metabolism [37]. Tuomainen et al. indicated that SF levels were associated with cholesterol oxidation products [38]. Premenopausal women, even with hypercholesterolemia, appeared to be protected against cardiovascular diseases [39]. However, the protective effect was absent in postmenopausal women, indicating a potential influence of iron status [39]. The results of thsese studies consistently suggested that the adverse effects of cholesterol could be influenced by iron status. Although SF has been proven to be an independent predictor of the development of DM [17, 34], previous studies have not focused on the effect of RC to DM in relation to iron status. Our study addressed this gap and also confirmed the findings of previous research. It was observed in our study that iron status played a mediating role in the relationship between RC and DM, indicating that abnormal iron metabolism may be employed as potential pathway in association between RC and DM. To our best knowledge, this is the first study to consider the mediation effect of iron status on that association.

The mechanism underlying the correlation between RC and iron status is not clear. Iron deficiency can affect the function of many liver enzymes involved in cholesterol metabolism [40]. The interrelationship between cholesterol levels and iron status has been confirmed in several experimental studies [41, 42]. For example, animal work with rats have found that an iron-adequate diet resulted in increased blood cholesterol levels [41]. Another study found that rats fed a high-fat diet accumulated more hepatic iron, compared with those fed regular diet [42]. Regarding the influence of iron status on cholesterol metabolism, studies have shown that iron levels were associated with inducing oxidative stress, lipid peroxidation, and aggravating insulin resistance [43, 44]. Iron status could alter lipid homeostasis by inducing sterol regulatory element-binding protein 2-mediated cholesterol biosynthesis, thus increasing their susceptibility to apoptosis [45]. All these biological effects can lead to β-cell apoptosis, resulting in defects in insulin synthesis and further contributing to the development of DM [5]. One study found that cholesterol plays a significant role in iron-dependent oxidative damage in neurodegenerative changes [46]. Interestingly, in our study, we observed that higher levels of RC were associated with increased levels of WBC, hs-CRP, and HOMA-IR, which suggested that inflammation and insulin resistance might play a role in the context of high RC and high iron status [47, 48], although oxidative markers were not measured in the present study for comparison. Additionally, in patients with DM, the expression of circulating miR-146a was found to be positively correlated with cholesterol and negatively with iron status, suggesting the complex regulation in these association at the genetic level [49]. In general, the effect of iron status on the pathogenicity of RC requires further evaluation.

## Strength and limitation

The major strength of this study was that we used of data from a nationally longitudinal survey in China with a large sample size to clarify the dose–response relationship between RC and iron status markers, which provides great statistical power. On the other hand, we explored the mediation effect of iron status between the relationship between RC and DM in population level. The present study had several limitations. First, the cross-sectional study could not determine the causality between RC and increased DM risk. Second, since the CHNS did not provide information on family history of cardiovascular diseases or medication about lipid-lowering therapy, iron supplementation, or iron-lowering therapy, we were unable to make further adjustments for these variables in our analysis. Third, in the current study we calculated the concentration of the RC by total cholesterol minus LDL-C minus HDL-C, which is not that accurate compared with the direct approach like ultracentrifugation or nuclear magnetic resonance spectroscopy. While until now, no study has identified directly measured or calculated RC best predicts DM risk. Finally, while we were able to incorporate much self-reported dietary information into analysis, we were not able to adjust for detailed food intake information focused specifically on iron intake.

## Conclusion

In conclusion, serum RC levels were significantly associated with iron status and risk of prevalent DM. Our study highlighted the mediating role of iron status in relation between RC and DM. Understanding the association and interaction between RC and iron load can help enhance the management of DM risk. Further investigations are warranted to corroborate our findings and determine the underlying mechanisms.

### Supplementary Information

**Additional file 1: Table S1.** Baseline characteristics of excluded and included participants. **Table S2.** Subgroup analysis for the associations between remnant cholesterol and SF. **Table S3.** Subgroup analysis for the associations between remnant cholesterol and TBI. **Table S4.** General linear regression models for the associations between remnant cholesterol and iron status considering TG. **Table S5.** General linear regression models for the associations between remnant cholesterol and iron status considering hs-CRP. **Table S6.** General linear regression models for the associations between remnant cholesterol and iron status. **Figure S1.** Directed acyclic graph of the association between remnant cholesterol and iron status. **Figure S2.** Directed acyclic graph of the association between remnant cholesterol and iron status. **Figure S3.** Mediation analysis of the association between RC and DM.

## Data Availability

The data used in this study can be available at https://www.cpc.unc.edu/projects/china.

## Abbreviations

BMI: Body mass index
CHNS: China Health and Nutrition Survey
CI: Confidence interval
CKD-EPI: Chronic Kidney Disease Epidemiology Collaboration
DAG: Directed acyclic graphs
DM: Diabetes mellitus
eGFR: Estimated glomerular filtration rate
FBG: Fasting blood glucose
HDL-C: High-density lipoprotein cholesterol
HOMA-IR: Homeostatic model assessment of insulin resistance
Hs-CRP: High-sensitivity C-reactive protein
IQR: Interquartile range
LDL-C: Low-density lipoprotein cholesterol
OR: Odds ratio
RC: Remnant cholesterol
RCS: Restricted cubic spline
SE: Standard error
SF: Serum ferritin
sTfR: Soluble transferrin receptor
TBI: Total body iron
TC: Total cholesterol
TG: Triglyceride
TRLs: Triglyceride-rich lipoproteins
WBCs: White blood cells
