# Supplementary material for: Remnant cholesterol, iron status and diabetes mellitus: a dose–response relationship and mediation analysis
Source: Diabetol Metab Syndr. 2024 Mar 12;16:65. doi: 10.1186/s13098-024-01304-0 (PMC10929145; doi:10.1186/s13098-024-01304-0)
Supplement: Supplementary file 1 — Additional file 1: Table S1. Baseline characteristics of excluded and included participants. Table S2. Subgroup analysis for the associations between remnant cholesterol and SF. Table S3. Subgroup analysis for the associations between remnant cholesterol and TBI. Table S4. General linear regression models for the associations between remnant cholesterol and iron status considering TG. Table S5. General linear regression models for the associations between remnant cholesterol and iron status considering hs-CRP. Table S6. General linear regression models for the associations between remnant cholesterol and iron status. Figure S1. Directed acyclic graph of the association between remnant cholesterol and iron status. Figure S2. Directed acyclic graph of the association between remnant cholesterol and iron status. Figure S3. Mediation analysis of the association between RC and DM. [file 13098_2024_1304_MOESM1_ESM.docx]

**Remnant cholesterol, iron status and diabetes mellitus: a dose-response relationship and mediation analysis**

Additional file 1: Table S1. Baseline characteristics of excluded and included participants

Additional file 1: Table S2. Subgroup analysis for the associations between remnant cholesterol and SF

Additional file 1: Table S3. Subgroup analysis for the associations between remnant cholesterol and TBI

Additional file 1: Table S4. General linear regression models for the associations between remnant cholesterol and iron status considering TG

Additional file 1: Table S5. General linear regression models for the associations between remnant cholesterol and iron status considering hs-CRP

Additional file 1: Table S6. General linear regression models for the associations between remnant cholesterol and iron status

Additional file 1: Figure S1. Directed acyclic graph of the association between remnant cholesterol and iron status

Additional file 1: Figure S2. Directed acyclic graph of the association between remnant cholesterol and iron status

Additional file 1: Figure S3. Mediation analysis of the association between RC and DM

Additional file 1: Table S1. Baseline characteristics of excluded and included participants

|  | Excluded  N = 1864 | Included  N = 7685 |
| --- | --- | --- |
| Age, year | 32.48 ± 22.07 | 50.63 ± 15.16 |
| BMI, kg/m^2^ | 20.42 ± 4.03 | 23.47 ± 3.47 |
| Male | 864 (46.35%) | 3675 (47.82%) |
| High school or above | 271 (14.54%) | 1838 (23.92%) |
| Residence |  |  |
| Urban | 541 (29.02%) | 2544 (33.10%) |
| Rural | 1323 (70.98%) | 5141 (66.90%) |
| Smoking | 274 (14.70%) | 2413 (31.40%) |
| Alcohol consumption | 149 (7.99%) | 1006 (13.09%) |

*BMI*, Body mass index

Additional file 1: Table S2. Subgroup analysis for the associations between remnant cholesterol and SF

| Sub-group | n | β (95% CI) | p for interaction |
| --- | --- | --- | --- |
| Age, year |  |  | 0.569 |
| ＜60 | 5636 | 83.84 (76.05—91.63) |  |
| ≥60 | 2049 | 78.85 (64.86—92.83) |  |
| Sex |  |  | <0.001 |
| Male | 3675 | 89.15 (78.76—99.56) |  |
| Female | 4010 | 46.12 (39.30—52.94) |  |
| BMI, kg/m^2^ |  |  | 0.977 |
| < 18 | 461 | 51.21 (17.19—85.22) |  |
| 18-24 | 4851 | 79.40 (70.52—88.27) |  |
| > 24 | 2373 | 75.82 (63.80—87.83) |  |
| Residence |  |  | 0.848 |
| Urban | 2544 | 83.69 (72.23—95.16) |  |
| Rural | 5141 | 82.28 (73.88—90.68) |  |
| Occupation |  |  | <0.001 |
| Farmer | 3805 | 65.30 (56.14—74.46) |  |
| Non-Farmer | 3880 | 96.59 (86.67—106.50) |  |
| Education |  |  | 0.001 |
| Below high school | 5855 | 75.28 (67.76—82.79) |  |
| High school or above | 1830 | 102.22 (87.16—117.27) |  |
| Smoking |  |  | 0.248 |
| No | 5272 | 75.88 (68.39—83.37) |  |
| Yes | 2413 | 84.00 (70.87—97.13) |  |
| Alcohol consumption |  |  | 0.303 |
| No | 6679 | 77.72 (68.37—84.89) |  |
| Yes | 1006 | 86.45 (67.39—105.51) |  |
| Chronic kidney disease |  |  | 0.540 |
| No | 6755 | 83.26 (76.08—90.45) |  |
| Yes | 930 | 75.42 (54.49—96.34) |  |
| Anemia |  |  | 0.504 |
| No | 7280 | 81.74 (74.78— 88.70) |  |
| Yes | 405 | 93.26 (63.89—122.62) |  |

*BMI*, Body mass index

Additional file 1: Table S3. Subgroup analysis for the associations between remnant cholesterol and TBI

| Sub-group | n | β (95% CI) | p for interaction |
| --- | --- | --- | --- |
| Age, year |  |  | 0.008 |
| ＜60 | 5636 | 2.04 (1.85—2.24) |  |
| ≥60 | 2049 | 1.47 (1.17—1.77) |  |
| Sex |  |  | 0.056 |
| Male | 3675 | 1.49 (1.32—1.66) |  |
| Female | 4010 | 1.80 (1.53—2.08) |  |
| BMI, kg/m^2^ |  |  | 0.003 |
| < 18 | 461 | 1.31 (-0.02—2.64) |  |
| 18-24 | 4851 | 2.10 (1.86—2.34) |  |
| > 24 | 2373 | 1.49 (1.25—1.74) |  |
| Residence |  |  | 0.425 |
| Urban | 2544 | 2.00 (1.72—2.28) |  |
| Rural | 5141 | 1.86 (1.65—2.06) |  |
| Occupation |  |  | 0.026 |
| Farmer | 3805 | 1.69 (1.45—1.93) |  |
| Non-farmer | 3880 | 2.07 (1.84—2.30) |  |
| Education |  |  | <0.001 |
| Below high school | 5855 | 1.72 (1.52—1.91) |  |
| High school or above | 1830 | 2.42 (2.10—2.74) |  |
| Smoking |  |  | 0.109 |
| No | 5272 | 1.88 (1.66—2.09) |  |
| Yes | 2413 | 1.61 (1.39—1.83) |  |
| Alcohol consumption |  |  | 0.010 |
| No | 6679 | 1.92 (1.73—2.11) |  |
| Yes | 1006 | 1.39 (1.09—1.69) |  |
| Chronic kidney disease |  |  | 0.098 |
| No | 6755 | 1.95 (1.77—2.13) |  |
| Yes | 930 | 1.43 (0.95—1.91) |  |
| Anemia |  |  | 0.025 |
| No | 7280 | 1.82 (1.66—1.97) |  |
| Yes | 405 | 2.74 (1.43—4.05) |  |

*BMI*, Body mass index

Additional file 1: Table S4. General linear regression models for the associations between remnant cholesterol and iron status considering TG

|  | β-coefficients (95% CI) |
| --- | --- |
| SF, ng/ml |  |
| Each 1 mmol/L increase in RC | 47.88 (33.11—62.65) |
| Q1 (0.01—0.19) | Reference |
| Q2 (0.20—0.36) | 10.29 (-0.87—21.45) |
| Q3 (0.37—0.65) | 24.99 (13.37—36.60) |
| Q4 (0.66—8.68) | 34.86 (20.15—49.57) |
| P for trend | <0.001 |
| TBI, mg/kg |  |
| Each 1 mmol/L increase in RC | 1.20 (0.86—1.54) |
| Q1 (0.01—0.19) | Reference |
| Q2 (0.20—0.36) | 0.48 (0.22—0.73) |
| Q3 (0.37—0.65) | 0.89 (0.63—1.16) |
| Q4 (0.66—8.68) | 1.20 (0.86—1.53) |
| P for trend | <0.001 |

Model adjusted for age, sex, BMI, residence, occupation, education, smoking, alcohol consumption, eGFR, LDL-C, HDL-C, average energy intake, average carbohydrate intake, average fat intake, average protein intake, and TG.

*RC* Remnant cholesterol, *SF* Serum ferritin, *TBI* Total body iron

Additional file 1: Table S5. General linear regression models for the associations between remnant cholesterol and iron status considering hs-CRP

|  | β-coefficients (95% CI) |
| --- | --- |
| SF, ng/ml |  |
| Each 1 mmol/L increase in RC | 72.59 (65.26—79.93) |
| Q1 (0.01—0.19) | Reference |
| Q2 (0.20—0.36) | 14.52 (3.28—25.77) |
| Q3 (0.37—0.65) | 37.44 (25.88—49.01) |
| Q4 (0.66—8.68) | 85.08 (72.39—97.76) |
| P for trend | <0.001 |
| TBI, mg/kg |  |
| Each 1 mmol/L increase in RC | 1.60 (1.43—1.77) |
| Q1 (0.01—0.19) | Reference |
| Q2 (0.20—0.36) | 0.56 (0.31—0.82) |
| Q3 (0.37—0.65) | 1.14 (0.88—1.40) |
| Q4 (0.66—8.68) | 2.18 (1.89—2.47) |
| P for trend | <0.001 |

Model adjusted for age, sex, BMI, residence, occupation, education, smoking, alcohol consumption, eGFR, LDL-C, HDL-C, average energy intake, average carbohydrate intake, average fat intake, average protein intake, and hs-CRP.

*RC* Remnant cholesterol, *SF* Serum ferritin, *TBI* Total body iron

Additional file 1: Table S6. General linear regression models for the associations between remnant cholesterol and iron status

|  | Model 1 | Model 2 | Model 3 |
| --- | --- | --- | --- |
| SF, ng/ml | | | |
| Each 1 mmol/L increase in RC | 81.42 (74.57—88.28) | 64.95 (58.27—71.63) | 71.69 (64.27—79.11) |
| Q1 (0.01—0.19) | Reference | Reference | Reference |
| Q2 (0.20—0.36) | 18.86 (6.90—30.83) | 15.42 (4.13—26.71) | 14.96 (3.59—26.33) |
| Q3 (0.37—0.65) | 50.88 (38.95—62.80) | 38.66 (27.32—50.01) | 37.69 (26.01—49.37) |
| Q4 (0.66—8.68) | 112.45 (100.42—124.47) | 85.21 (73.46—96.97) | 85.30 (72.46—98.14) |
| P for trend | <0.001 | <0.001 | <0.001 |
| TBI, mg/kg | | | |
| Each 1 mmol/L increase in RC | 1.88 (1.71—2.05) | 1.39 (1.24—1.55) | 1.57 (1.40—1.74) |
| Q1 (0.01—0.19) | Reference | Reference | Reference |
| Q2 (0.20—0.36) | 0.68 (0.38—0.97) | 0.57 (0.32—0.83) | 0.57 (0.31—0.83) |
| Q3 (0.37—0.65) | 1.54 (1.25—1.83) | 1.17 (0.91—1.43) | 1.14 (0.87—1.41) |
| Q4 (0.66—8.68) | 2.93 (2.63—3.22) | 2.12 (1.85—2.39) | 2.16 (1.86—2.45) |
| P for trend | <0.001 | <0.001 | <0.001 |

Model 1: unadjusted;

Model 2: adjusted for age, sex and BMI;

Model 3: adjusted for age, sex, BMI, residence, occupation, education, smoking, alcohol consumption, eGFR, LDL-C, HDL-C, average energy intake, average carbohydrate intake, average fat intake, and average protein intake.

*RC* Remnant cholesterol, *SF* Serum ferritin, *TBI* Total body iron

Additional file 1: Figure S1. Directed acyclic graph of the association between remnant cholesterol and iron status


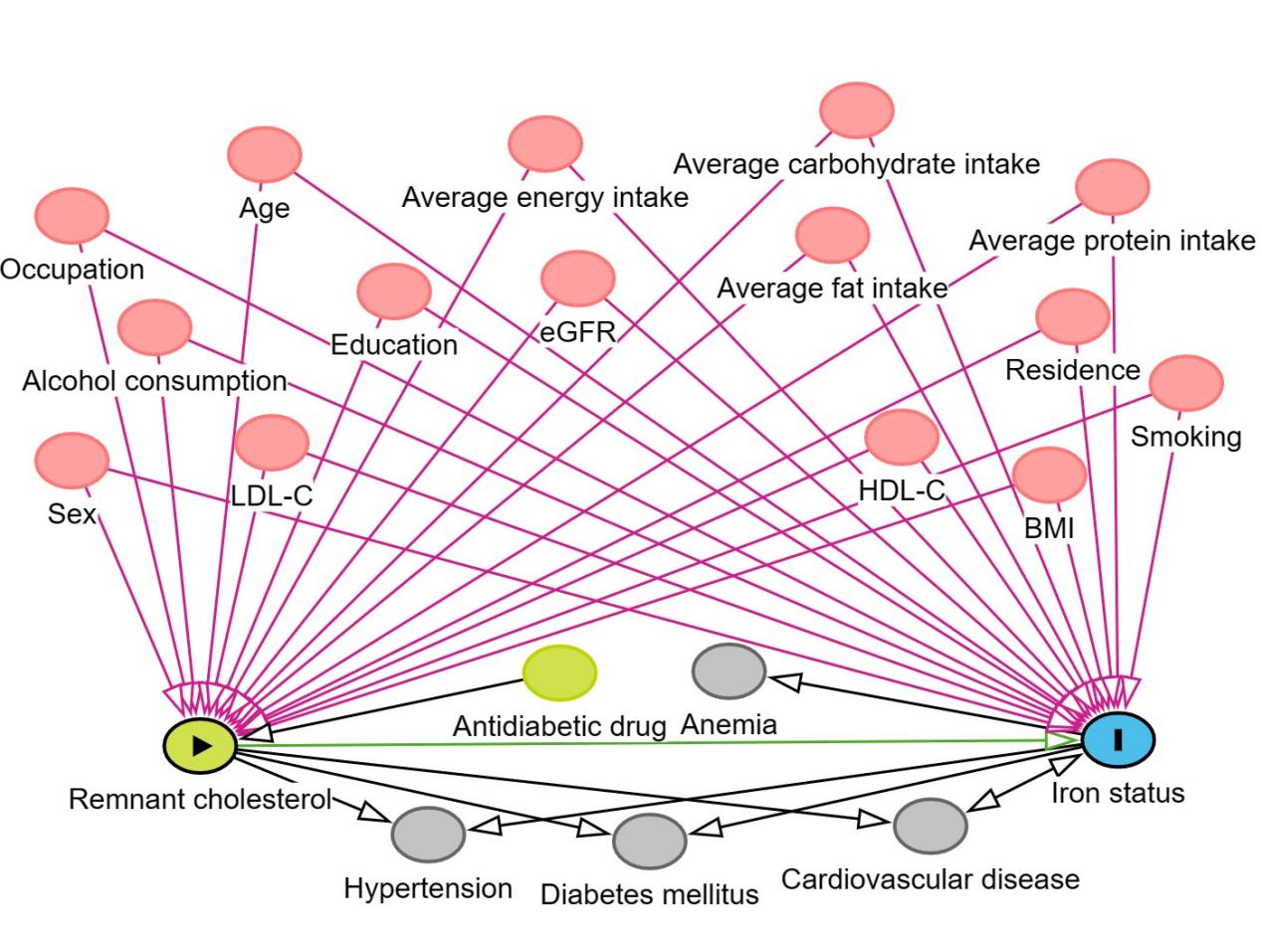


*BMI* Body mass index, *eGFR* Estimated glomerular filtration rate, *HDL-C* High-density lipoprotein cholesterol, *LDL-C* Low-density lipoprotein cholesterol

Additional file 1: Figure S2. Directed acyclic graph of the association between remnant cholesterol and iron status


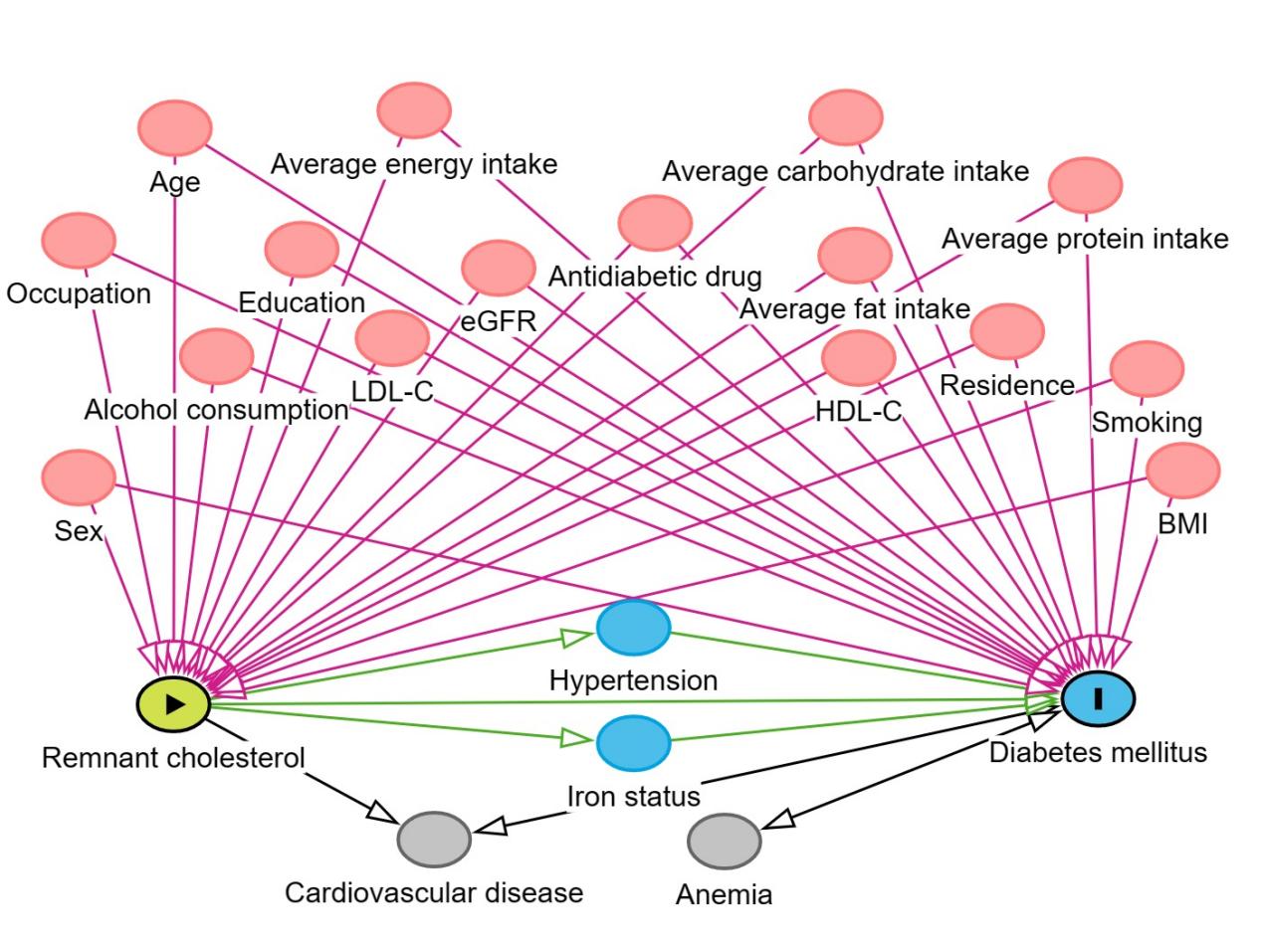


*BMI* Body mass index, *eGFR* Estimated glomerular filtration rate, *HDL-C* High-density lipoprotein cholesterol, *LDL-C* Low-density lipoprotein cholesterol

Additional file 1: Figure S3. Mediation analysis of the association between RC and DM


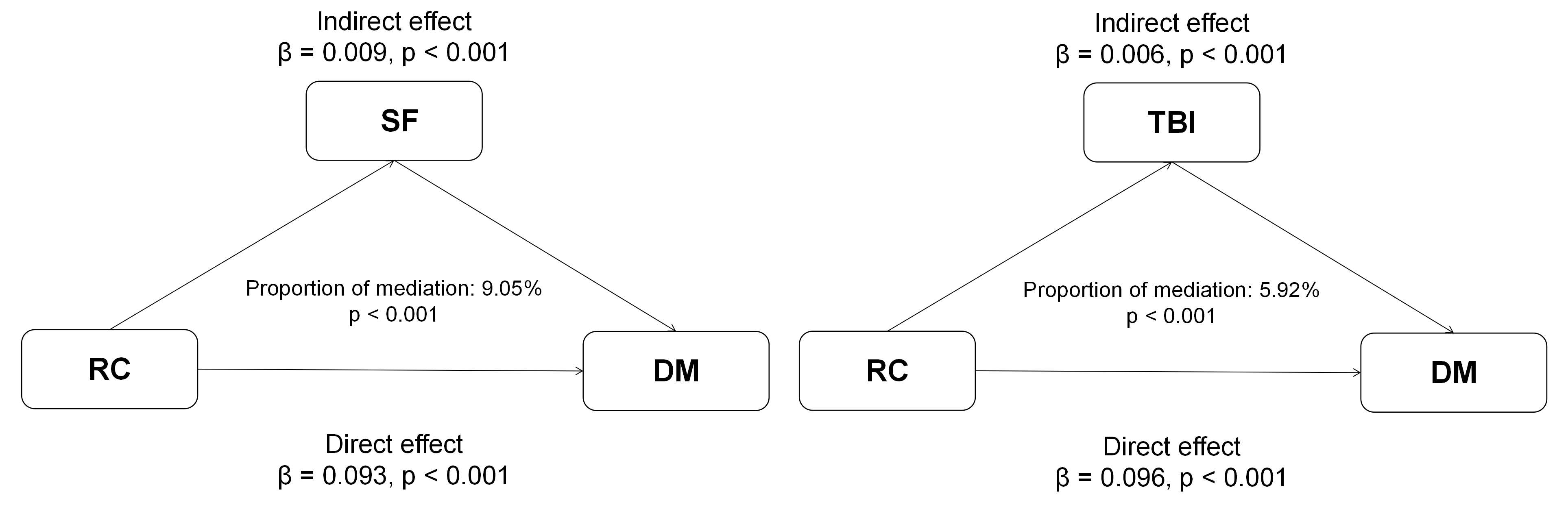


*DM* Diabetes mellitus, *RC* Remnant cholesterol, *SF* Serum ferritin, *TBI* Total body iron
